# Supplementary material for: Experimental evidence of Willis coupling in a one-dimensional effective material element
Source: Nat Commun. 2017 Jun 13;8:15625. doi: 10.1038/ncomms15625 (PMC5474687; doi:10.1038/ncomms15625)
Supplement: Supplementary Information — Supplementary Notes and Supplementary References. [file ncomms15625-s1.pdf]

SUPPLEMENTARY NOTE 1

Consider a function $f(x')$ which is analytic for x' in the domain $\Omega = (x - \Delta x, x + \Delta x)$ (that is, $f(x')$ is equal to its Taylor series expansion for $x' \in \Omega$). Then, the volume average of f over Ω may be written as

$$\begin{aligned}\langle f \rangle &= \frac{1}{2\Delta x}\int_{x-\Delta x}^{x+\Delta x} f(x')\mathrm{d}x', \\ &= \frac{1}{2\Delta x}\int_{x-\Delta x}^{x+\Delta x} \sum_{n=0}^{\infty} \frac{(x'-x'')^n}{n!}\frac{\partial^n}{\partial x''^n} f(x'')\mathrm{d}x', \\ &= \sum_{n=0}^{\infty} \frac{[(x-x'')+\Delta x]^{n+1} - [(x-x'')-\Delta x]^{n+1}}{2\Delta x(n+1)!}\frac{\partial^n}{\partial x''^n} f(x''), \end{aligned} \tag{1}$$

where x'' is some point in Ω. Assume there is a characteristic length scale k^{-1} such that $\partial^n f(x'')/\partial x''^n \sim k^n$. Then, the nth term of the summation in supplementary equation (1) is $O[(k\Delta x)^n]$, where $k\Delta x < 1$ because $f(x')$ is analytic over Ω. Thus the volume average of f may be written as

$$\langle f \rangle = f(x'') + (x - x'')\frac{\partial}{\partial x''} f(x'') + \frac{1}{2}\left((x-x'')^2 + \frac{\Delta x^2}{3}\right)\frac{\partial^2}{\partial x''^2} f(x'') + O\left[(k\Delta x)^3\right]. \tag{2}$$

Since each term is of higher order of $k\Delta x$ than the previous term, regular perturbation theory may be used to invert supplementary equation (2). First, note that $\langle f \rangle = f(x'') + O[k\Delta x]$, so $f(x'') = \langle f \rangle + O[k\Delta x]$ and, since f is arbitrary, $\partial f(x'')/\partial x'' = \langle \partial f/\partial x \rangle + O[k\Delta x]$. Substituting this expression into supplementary equation (2) truncated at second order yields

$$\langle f \rangle = f(x'') + (x - x'')\left\langle \frac{\partial f}{\partial x} \right\rangle + O\left[(k\Delta x)^2\right], \tag{3}$$

and so

$$f(x'') = \langle f \rangle - (x - x'')\left\langle \frac{\partial f}{\partial x} \right\rangle + O\left[(k\Delta x)^2\right], \tag{4}$$

$$\frac{\partial}{\partial x''} f(x'') = \left\langle \frac{\partial f}{\partial x} \right\rangle - (x - x'')\left\langle \frac{\partial^2 f}{\partial x^2} \right\rangle + O\left[(k\Delta x)^2\right]. \tag{5}$$

Again substituting these expressions into supplementary equation (2) and inverting yields

$$f(x'') = \langle f \rangle + (x'' - x)\left\langle \frac{\partial f}{\partial x} \right\rangle + \frac{1}{2}\left((x''-x)^2 - \frac{\Delta x^2}{3}\right)\left\langle \frac{\partial^2 f}{\partial x^2} \right\rangle + O\left[(k\Delta x)^3\right]. \tag{6}$$

In principle, this inversion process may be extended to arbitrary order, but the $O[(k\Delta x)^3]$ expressions in supplementary equations (2) and (6) will be sufficient for this work.

For convenience, the special case of $x'' = x$ yields the simpler expressions

$$f(x) = \langle f \rangle - \frac{\Delta x^2}{6} \left\langle \frac{\partial^2 f}{\partial x^2} \right\rangle + O\left[(k\Delta x)^3\right], \tag{7}$$

$$\langle f \rangle = f(x) + \frac{\Delta x^2}{6} \frac{\partial^2}{\partial x^2} f(x) + O\left[(k\Delta x)^3\right]. \tag{8}$$

SUPPLEMENTARY NOTE 2

The volume average momentum density of a one-dimensional material element may be written as

$$\langle \mu \rangle = \frac{1}{2\Delta x} \int_{x-\Delta x}^{x+\Delta x} \rho(x') v(x') \mathrm{d}x', \tag{9}$$

where $2\Delta x$ is the length of the element and x is the position of the center of the element. Both $\mu(x)$ and $v(x')$ depend on both space and (implicitly) time. In general, the integral in supplementary equation (9) must be performed numerically, since $\rho(x')$ and $v(x')$ need not be analytic functions. However, treating a system as a material inherently assumes the associated acoustic fields vary *continuously*, which is a good approximation for $(k\Delta x) = k\Delta x \ll 1$, where k is the largest wavenumber of interest. While low-frequency excitations guarantee $k\Delta x \ll 1$ for the acoustic fields (for example, v and μ), this is not the case for the constituent material properties (for example, ρ). In the present derivation, we will restrict ourselves to systems where the microstructural properties vary smoothly and slowly, such that $k\Delta x \ll 1$, where k is the largest spatial frequency of the microstructure. While this restriction is not necessary from a practical standpoint, it allows us to present results in an easily interpreted form. Then, under the continuous material approximation and using the results from the section above, both $\rho(x')$ and $v(x')$ may be expanded as

$$f(x') = \langle f \rangle + \left\langle \frac{\partial f}{\partial x} \right\rangle (x' - x) + \frac{1}{2} \left\langle \frac{\partial^2 f}{\partial x^2} \right\rangle \left((x' - x)^2 - \frac{\Delta x^2}{3} \right) + O\left[(k\Delta x)^3\right], \tag{10}$$

for $f \in \{\rho, v\}$. Thus, the integrand of supplementary equation (9) may be written as

$$\begin{aligned} \rho(x')v(x') = \langle \rho \rangle \langle v \rangle + \left[\langle \rho \rangle \left\langle \frac{\partial v}{\partial x} \right\rangle + \left\langle \frac{\partial \rho}{\partial x} \right\rangle \langle v \rangle \right] (x' - x) + \\ + \frac{1}{2} \left[2 \left\langle \frac{\partial \rho}{\partial x} \right\rangle \left\langle \frac{\partial v}{\partial x} \right\rangle + \langle \rho \rangle \left\langle \frac{\partial^2 v}{\partial x^2} \right\rangle + \left\langle \frac{\partial^2 \rho}{\partial x^2} \right\rangle \langle v \rangle \right] (x' - x)^2 \\ - \frac{\Delta x^2}{6} \left[\langle \rho \rangle \left\langle \frac{\partial^2 v}{\partial x^2} \right\rangle + \left\langle \frac{\partial^2 \rho}{\partial x^2} \right\rangle \langle v \rangle \right] + O\left[(k\Delta x)^2\right]. \end{aligned} \tag{11}$$

The integration of supplementary equation (9) may now be performed, yielding

$$\langle \mu \rangle = \langle \rho \rangle \langle v \rangle + \frac{\Delta x^2}{3} \left\langle \frac{\partial \rho}{\partial x} \right\rangle \left\langle \frac{\partial v}{\partial x} \right\rangle + O\left[(k\Delta x)^3\right]. \quad (12)$$

Further, making use of the continuous acoustic field approximation, the momentum, velocity, and velocity gradient may be expanded using

$$\langle g \rangle = g(x) + \frac{\Delta x^2}{6} \frac{\partial^2}{\partial x^2} g(x) + O\left[(k\Delta x)^3\right], \quad (13)$$

for $g \in \{\mu, v, \partial v/\partial x\}$. Thus supplementary equation (12) may be written as

$$\mu(x) + \frac{\Delta x^2}{6} \frac{\partial^2}{\partial x^2} \mu(x) = \langle \rho \rangle v(x) + \frac{\Delta x^2}{3} \left\langle \frac{\partial \rho}{\partial x} \right\rangle \frac{\partial}{\partial x} v(x) + \frac{\Delta x^2}{6} \langle \rho \rangle \frac{\partial^2}{\partial x^2} v(x) + O\left[(k\Delta x)^3\right]. \quad (14)$$

Note that supplementary equation (9) shows that to $O[k\Delta x]$ the momentum density may be written as

$$\mu(x) = \langle \rho \rangle \langle v \rangle + O[k\Delta x]. \quad (15)$$

Then, substituting supplementary equation (15) into the left-hand side of supplementary equation (14) and simplifying yields

$$\mu(x) = \langle \rho \rangle v(x) + \frac{\Delta x^2}{3} \left\langle \frac{\partial \rho}{\partial x} \right\rangle \frac{\partial}{\partial x} v(x) + O\left[(k\Delta x)^3\right]. \quad (16)$$

Similarly, the average volumetric strain of the one-dimensional fluid element may be written to $O[(k\Delta x)^3]$ as

$$\langle \varepsilon \rangle = -\frac{1}{2\Delta x} \int_{x-\Delta x}^{x+\Delta x} \kappa^{-1}(x') p(x') \mathrm{d}x', \quad (17)$$

and using parallel arguments to the discussion of the average momentum density given above, the volumetric strain field may be written as

$$\varepsilon(x) = -\langle \kappa^{-1} \rangle p(x) - \frac{\Delta x^2}{3} \left\langle \frac{\partial (\kappa^{-1})}{\partial x} \right\rangle \frac{\partial}{\partial x} p(x) + O\left[(k\Delta x)^3\right]. \quad (18)$$

Though not in standard form, supplementary equations (16) and (18) are constitutive equations for the momentum density and volumetric strain of the material element, respectively. Using the expression of conservation of momentum for fluid elements, $-\langle \partial p/\partial x \rangle = \partial \langle \mu \rangle / \partial t$ (or $\partial p/\partial x = \partial \mu/\partial t + O[(k\Delta x)^2]$, using the results of the section above), and the definition of the strain rate, $\partial \varepsilon/\partial t = \partial v/\partial x$, supplementary equations (16) and (18) may be rearranged to yield familiar forms of the constitutive relations including higher order effects

to $O[(k\Delta x)^2]$

$$\mu(x) = \langle\rho\rangle v(x) + \frac{\Delta x^2}{3}\left\langle\frac{\partial\rho}{\partial x}\right\rangle\frac{\partial}{\partial t}\varepsilon(x) + O\left[(k\Delta x)^3\right], \tag{19}$$

$$-p(x) = \frac{1}{\langle\kappa^{-1}\rangle}\varepsilon(x) - \frac{\Delta x^2}{3}\frac{\langle\rho\rangle}{\langle\kappa^{-1}\rangle}\left\langle\frac{\partial(\kappa^{-1})}{\partial x}\right\rangle\frac{\partial}{\partial t}v(x) + O\left[(k\Delta x)^3\right]. \tag{20}$$

By truncating the expressions at $O[(k\Delta x)^2]$ and matching terms in supplementary equations (19) and (20) with the assumed constitutive relationship for Willis media in Eqs. (1) of the main text, the effective mass density and bulk modulus of the element may be written as

$$\rho^{\text{eff}} = \langle\rho\rangle \quad \text{and} \quad \kappa^{\text{eff}} = \frac{1}{\langle\kappa^{-1}\rangle}, \tag{21}$$

respectively. This is consistent with the usual use of arithmetic and harmonic averages to estimate the effective mass density and bulk modulus, respectively, of heterogeneous fluids (emulsions) and suspensions. Further, the second terms on the right-hand side of supplementary equations (19) and (20) can be identified as the Willis coupling coefficients:

$$\tilde{\psi} = \frac{\Delta x^2}{3}\left\langle\frac{\partial\rho}{\partial x}\right\rangle \quad \text{and} \quad \psi = -\frac{\Delta x^2}{3}\frac{\langle\rho\rangle}{\langle\kappa^{-1}\rangle}\left\langle\frac{\partial(\kappa^{-1})}{\partial x}\right\rangle. \tag{22}$$

For linear, reciprocal systems the ratio of $\tilde{\psi}$ and ψ in supplementary equation (22) must be equal at $O[k\Delta x]$ (since the terms involved in the constitutive equations are already $O[(k\Delta x)^2]$), or

$$-\frac{\langle\rho\rangle}{\langle\kappa^{-1}\rangle}\left\langle\frac{\partial(\kappa^{-1})}{\partial x}\right\rangle \Big/ \left\langle\frac{\partial\rho}{\partial x}\right\rangle = 1 + O\left[k\Delta x\right]. \tag{23}$$

By considering a small isolated element, it is acceptable to only keep terms $O[1]$ for ρ^{eff} and κ^{eff}, and terms $O[k\Delta x]$ for $\psi = \tilde{\psi}$. As demonstrated in Supplementary Note 2, material asymmetry is observed at $O[(k\Delta x)^1]$, which is referred to here as local coupling, and is due to the microstructure. On the other hand, nonlocal coupling describes an artificial asymmetry introduced by finite effective phase velocity across the sample and multiple scattering.[1] As a result, nonlocal coupling occurs at $O[(k\Delta x)^2]$. Being able to neglect nonlocal coupling significantly reduces the problem because including it would also require determining $O[(k\Delta x)^2]$ contributions in ρ^{eff}, κ^{eff} and ψ as well. Justification for neglecting nonlocal coupling in the sample studied in this work is provided by the good agreement between the lumped-element model and the measured properties in Fig. 5, since the lump-element model does not account for $O[(k\Delta x)^2]$.

While not a general proof, supplementary equation (23) may be shown to be true for lossless ideal gases with linear disturbances. The bulk modulus in lossless fluids may be defined as

$$\kappa = \rho \frac{\partial P}{\partial \rho}. \tag{24}$$

Note that for adiabatic processes in an ideal gas $P\rho^{-\gamma} = \text{constant}$, where γ is the adiabatic gas constant, and so taking the differential yields

$$\rho^{-\gamma} dP - \gamma \rho^{-\gamma-1} P d\rho = 0 \Rightarrow \frac{dP}{d\rho} = \gamma \frac{P}{\rho} = \gamma RT, \tag{25}$$

where R and T are the gas constant and temperature, respectively. The quantity γRT, which may be identified as the square of the linear sound speed c_0^2, depends only on the temperature. Thus, for linear oscillations (for which T does not depend on the acoustic field) and assuming uniform ambient temperature $\kappa(x) = c_0^2 \rho(x)$, and the left-hand side of supplementary equation (23) becomes

$$-\frac{\langle \rho \rangle}{c_0^{-2} \langle \rho^{-1} \rangle} c_0^{-2} \left\langle \frac{\partial(\rho^{-1})}{\partial x} \right\rangle \bigg/ \left\langle \frac{\partial \rho}{\partial x} \right\rangle = \frac{\langle \rho \rangle}{\langle \rho^{-1} \rangle} \left\langle \frac{1}{\rho^2} \frac{\partial \rho}{\partial x} \right\rangle \bigg/ \left\langle \frac{\partial \rho}{\partial x} \right\rangle. \tag{26}$$

Note that $\langle f^{-1} \rangle^{-1} = \langle f \rangle + O[(k\Delta x)^2]$, $\langle f^2 \rangle = \langle f \rangle^2 + O[k\Delta x]$, and $\langle fg \rangle = \langle f \rangle \langle g \rangle + O[(k\Delta x)^2]$, and so the left-hand side of supplementary equation (23) may be written as

$$\left[\frac{\langle \rho \rangle \left(\langle \rho \rangle + O[(k\Delta x)^2] \right)}{\langle \rho \rangle^2 + O[k\Delta x]} \left\langle \frac{\partial \rho}{\partial x} \right\rangle + O[(k\Delta x)^2] \right] \bigg/ \left\langle \frac{\partial \rho}{\partial x} \right\rangle = 1 + O[k\Delta x], \tag{27}$$

and so reciprocity holds.

SUPPLEMENTARY NOTE 3

We will use the notation of Fig. (1) of the main text. The background material is assumed to have a wavenumber k_0 and characteristic impedance Z_0, while the middle layer of length L has a wavenumber k, characteristic impedance Z and Willis coupling coefficient ψ. Thus, the middle layer's specific acoustic impedance in the $\pm x$ direction may be written as

$$Z_{\rm sp}^{\pm} = Z(\pm 1 + iW), \tag{28}$$

where $W = \omega\psi/Z$ is the asymmetry coefficient. Then, continuity of pressure at boundaries is written mathematically as

$$p_0(0) + p_1(0) = p_2(0) + p_3(0), \tag{29}$$

$$p_2(0)e^{ikL} + p_3(0)e^{-ikL} = p_4(L) + p_5(L), \tag{30}$$

and continuity of velocity at boundaries as

$$\frac{p_0(0)}{Z_0} - \frac{p_1(0)}{Z_0} = \frac{p_2(0)}{Z(1+iW)} - \frac{p_3(0)}{Z(1-iW)}, \tag{31}$$

$$\frac{p_2(0)e^{ikL}}{Z(1+iW)} - \frac{p_3(0)e^{-ikL}}{Z(1-iW)} = \frac{p_4(L)}{Z_0} + \frac{p_5(L)}{Z_0}. \tag{32}$$

Assuming an anechoic termination at $x \gg L$ (such that $p_5 = 0$), the forward reflection and transmission coefficients may be written as $R = p_1(0)/p_0(0)$ and $T = p_4(L)/p_0(0)$. On the other hand, if there is an anechoic termination located at $x \ll 0$ (such that $p_0 = 0$), the backward reflection and transmission coefficients may be written as $R_{\mathrm{B}} = p_4(L)/p_5(L)$ and $T_{\mathrm{B}} = p_1(0)/p_5(L)$. Defined in this manner, R, T, R_{B} and T_{B} and are sometimes referred to as scattering coefficients. Using these definitions, supplementary equations (29)–(32) may be summarized in matrix form as

$$\begin{bmatrix} 1 & -1 & -1 & 0 \\ -1 & -\frac{Z_0/Z}{1+iW} & \frac{Z_0/Z}{1-iW} & 0 \\ 0 & e^{ikL} & e^{-ikL} & -1 \\ 0 & \frac{Z_0/Z}{1+iW}e^{ikL} & -\frac{Z_0/Z}{1-iW}e^{-ikL} & -1 \end{bmatrix} \begin{bmatrix} R \\ p_2/p_0 \\ p_3/p_0 \\ T \end{bmatrix} = \begin{bmatrix} -1 \\ -1 \\ 0 \\ 0 \end{bmatrix}. \tag{33}$$

This may be solved for R and T (using, for instance, Kramer's rule)

$$R = \frac{(z^2(1+W^2)-1)\sin(kL) + 2iWz\sin(kL))}{(z^2\,(1+W^2)+1)\sin(kL) + 2iz\cos(kL)}, \tag{34}$$

$$T = \frac{2iz}{(z^2\,(1+W^2)+1)\sin(kL) + 2iz\cos(kL)}, \tag{35}$$

where $z = Z/Z_0$. Inverting the orientation of the effective material element would only change $\psi \to -\psi$, and so $W \to -W$. Thus, R_{B}, or the reflection coefficient in the inverted orientation, may be written as

$$R_{\mathrm{B}} = \frac{(z^2(1+W^2)-1)\sin(kL) - 2iWz\sin(kL))}{(z^2\,(1+W^2)+1)\sin(kL) + 2iz\cos(kL)}. \tag{36}$$

Supplementary equations (34), (35), and (36) may then be inverted for $\cos(kL)$, $Z = zZ_0$, and W as

$$\cos(kL) = \frac{1 - RR_{\mathrm{B}} + T^2}{2T}, \tag{37}$$

$$W = \pm\frac{R_{\mathrm{B}} - R}{\sqrt{4T^2 - (1 - RR_{\mathrm{B}} + T^2)^2}}, \tag{38}$$

and

$$Z = \pm\frac{Z_0}{\sqrt{1+W^2}}\sqrt{\frac{(1+R)(1+R_{\mathrm{B}}) - T^2}{(1-R)(1-R_{\mathrm{B}}) - T^2}}. \tag{39}$$

Notice that $\cos(kL)$ is 2π periodic in the phase kL, and so care must be taken in taking the inverse cosine. In the limit $kL \to 0$ we may simply write

$$k = \pm\frac{1}{L}\cos^{-1}\left(\frac{1 - RR_{\mathrm{B}} + T^2}{2T}\right), \tag{40}$$

where the $\pm$ acknowledges the fact that there may be a negative sound speed. If there are m full wavelengths present within the effective material element, then we may write

$$k = \pm\frac{1}{L}\cos^{-1}\left(\frac{1 - RR_{\mathrm{B}} + T^2}{2T}\right) \mp \frac{2\pi m}{L}. \tag{41}$$

As discussed by Fokin, *et al.*,[2] the signs of Z and k are not independent of each other. The choice of sign for Z and k are dictated by the fact that for a passive system the real part of Z must be positive. Then, following the method developed by Fokin, *et al.*, the effective impedance and wavenumber may be written as

$$Z = \frac{Z_0 r}{(1 - R)(1 - R_{\mathrm{B}}) - T^2} \tag{42}$$

$$\text{and} \qquad k = \frac{i\log(x)}{L} + \frac{2\pi m}{L}, \tag{43}$$

respectively, where $r = \pm\sqrt{(1 - RR_{\mathrm{B}} + T^2)^2 - 4T^2}$ and $x = (1 - RR_{\mathrm{B}} + T^2 + r)/2T$. Using these definitions, we can then write W in terms of r as

$$W = \pm\frac{R_{\mathrm{B}} - R}{ir}. \tag{44}$$

Using this approach, the sign of r is chosen such that the real part of Z is positive and we note that the value of m, which accounts for phase wrapping, is zero in the limit of quasi-static motion. The positive sign is chosen for W in the quasi-static limit, and at higher frequencies the sign is determined by requiring W be a continuous function of frequency. These are the equations referenced in the main text.

SUPPLEMENTARY NOTE 4

One aspect of using lumped-element analysis is that the dynamics of the system can be described using Newton's second law rather than wave equations. Thus, for the effective material element described in the main text, the positions of the boundaries satisfy the

equations

$$M_{\mathrm{m}}\frac{\partial^2 x_{\mathrm{m}}}{\partial t^2} = F_{\mathrm{m}} - k_{\mathrm{a}}(x_{\mathrm{m}} - x_{\mathrm{h}}) - k_{\mathrm{m}}x_{\mathrm{m}}, \tag{45}$$

$$M_{\mathrm{h}}\frac{\partial^2 x_{\mathrm{h}}}{\partial t^2} = F_{\mathrm{h}} + k_{\mathrm{a}}(x_{\mathrm{m}} - x_{\mathrm{h}}), \tag{46}$$

where x_{m} and x_{h} are the displacements of the membrane and the air masses in the holes of the paper, respectively, and F_{m} and F_{h} are the external forces (integral of pressure over the surface area) acting on the membrane and air masses, respectively (see the schematic in Fig. (2) of the main text). Assuming time harmonic motion with angular frequency ω, these equations become algebraic and may be solved for the volume- and surface-averaged quantities in the effective material element $\langle f\rangle = (F_{\mathrm{m}} + F_{\mathrm{h}})/V$ and $-\langle p\rangle = (F_{\mathrm{h}} - F_{\mathrm{m}})/2S$, where $V = SL$ is the volume of the effective material element and L is the length of the element in the propagation direction. Supplementary equations (45) and (46) can then be re-arranged and written in terms of the volume-averaged displacement, $\langle x\rangle = (x_{\mathrm{m}} + x_{\mathrm{h}})/2$ and strain, $\langle \varepsilon\rangle = (x_{\mathrm{h}} - x_{\mathrm{m}})/L$, to yield

$$\langle f\rangle = \frac{1}{V}\left[k_{\mathrm{m}} - \omega^2(M_{\mathrm{m}} + M_{\mathrm{h}})\right]\langle x\rangle - \frac{1}{2S}\left[k_{\mathrm{m}} - \omega^2(M_{\mathrm{m}} - M_{\mathrm{h}})\right]\langle \varepsilon\rangle, \tag{47}$$

$$-\langle p\rangle = -\frac{1}{2S}\left[k_{\mathrm{m}} - \omega^2(M_{\mathrm{m}} - M_{\mathrm{h}})\right]\langle x\rangle + \frac{L}{4S}\left[k_{\mathrm{m}} + 4k_{\mathrm{a}} - \omega^2(M_{\mathrm{m}} + M_{\mathrm{h}})\right]\langle \varepsilon\rangle. \tag{48}$$

Since the averaging process is only a geometrical average, time derivatives of the averaged fields follow as usual: $\langle \partial v/\partial t\rangle = -i\omega\langle v\rangle = -\omega^2\langle x\rangle$ and $\langle \partial\varepsilon/\partial t\rangle = -i\omega\langle\varepsilon\rangle$. We then note that Eqs. (15)–(18) in the main text provide estimates for the effective properties in terms of the volume averaged quantities in Eqs. (47) and (48). Equations (15)–(18) from the main text and supplementary equations (47) and (48) therefore provide the following estimates of the effective properties of the homogenized element, which includes Willis coupling,

$$\rho^{\mathrm{eff}} = -\frac{k_{\mathrm{m}}}{\omega^2 V} + \frac{M_{\mathrm{m}} + M_{\mathrm{h}}}{V}, \tag{49}$$

$$\kappa^{\mathrm{eff}} = \frac{L}{4S}(k_{\mathrm{m}} + 4k_{\mathrm{a}}) - \frac{\omega^2 L}{4S}(M_{\mathrm{m}} + M_{\mathrm{h}}) \tag{50}$$

and $$\psi^{\mathrm{eff}} = \tilde{\psi}^{\mathrm{eff}} = \frac{k_{\mathrm{m}}}{\omega^2 2S} - \frac{M_{\mathrm{m}} - M_{\mathrm{h}}}{2S}. \tag{51}$$

SUPPLEMENTARY REFERENCES

[1]A. Alù, "First-principles homogenization theory for periodic metamaterials," Phys. Rev. B **84**, 075153 (2011).

[2]V. Fokin, M. Ambati, C. Sun, and X. Zhang, "Method for retrieving effective properties of locally resonant acoustic metamaterials," Phys. Rev. B **76**, 144302 (2007).
